# Supplementary material for: Nitric Oxide Mediation in Hydroxyurea and Nitric Oxide Metabolites’ Inhibition of Erythroid Progenitor Growth
Source: Biomolecules. 2021 Oct 21;11(11):1562. doi: 10.3390/biom11111562 (PMC8616001; doi:10.3390/biom11111562)
Supplement: Supplementary file 1 [file biomolecules-11-01562-s001.zip › biomolecules-1377585-supplementary.pdf]

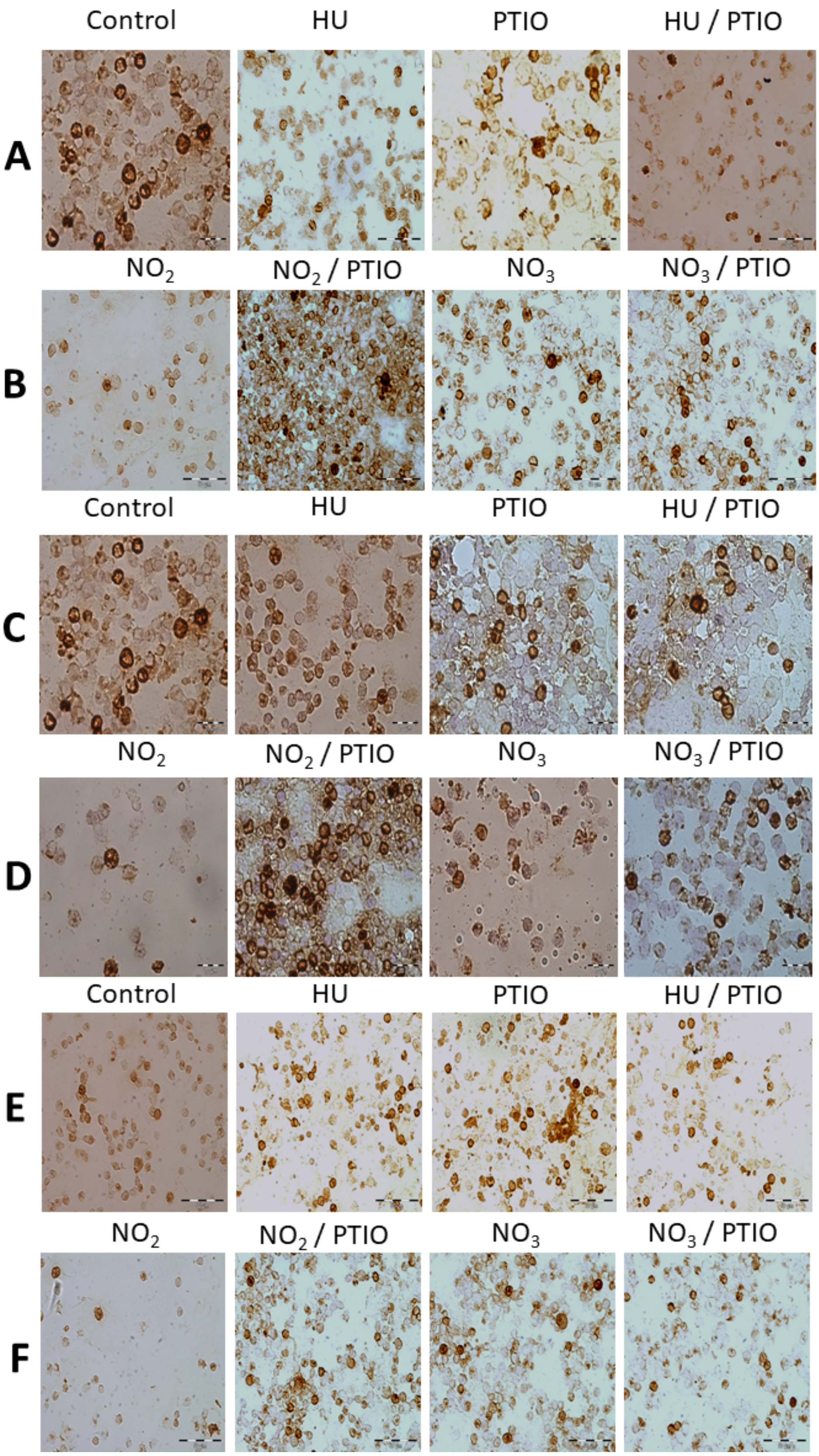

Figure S1. Immunocytochemistry slides correspond to Figure 4. Columns in graphs (**A**,**C**,**E**), while in graphs (**B**,**D**,**F**) correspond to NO_2, NO_2/PTIO, NO_3, NO_3/PTIO labelled columns.

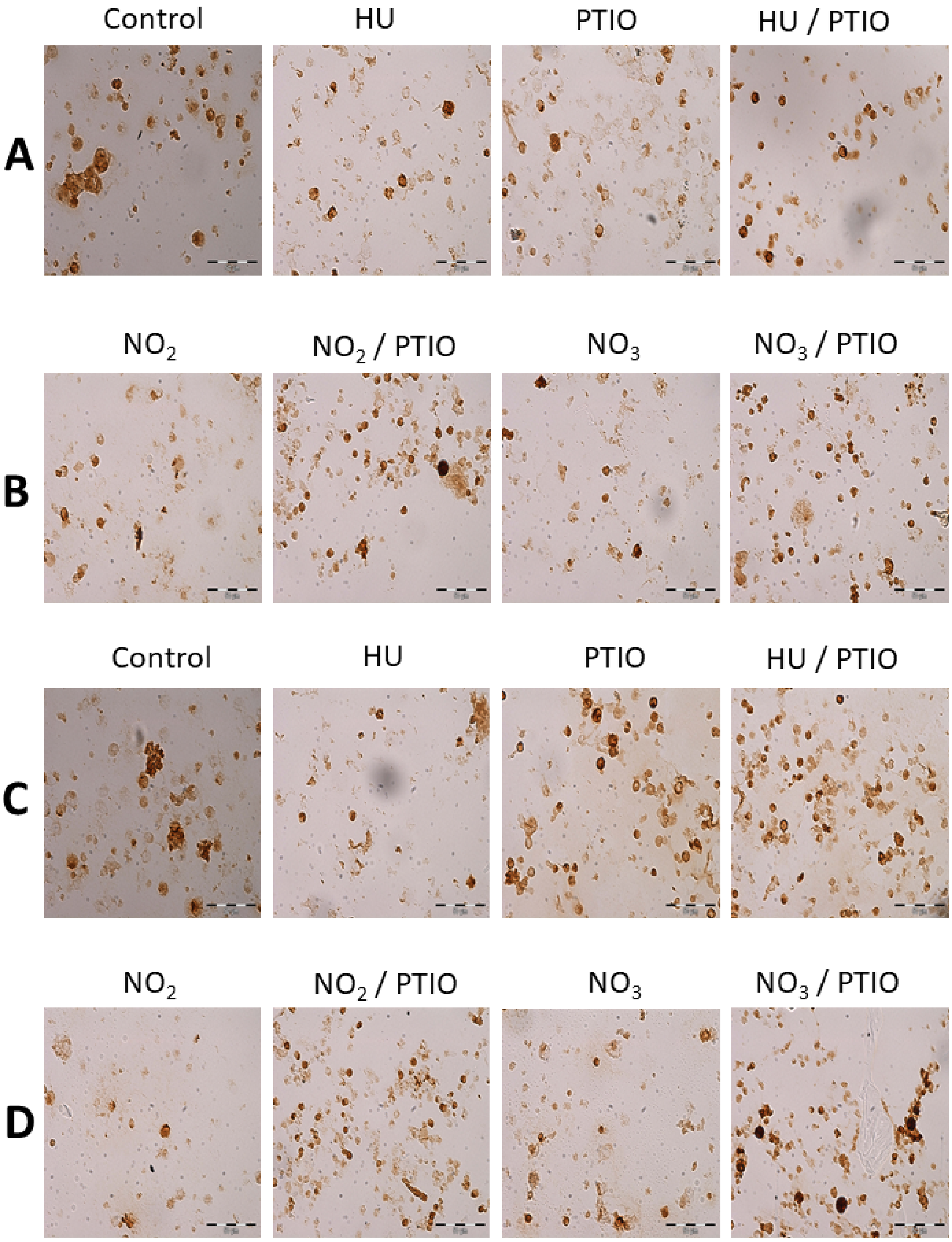

Figure S2. Immunocytochemistry slides correspond to Figure 5. Columns in graphs (**A**,**C**), while in graphs (**B**,**D**) correspond to NO_2, NO_2/PTIO, NO_3, NO_3/PTIO labelled columns.

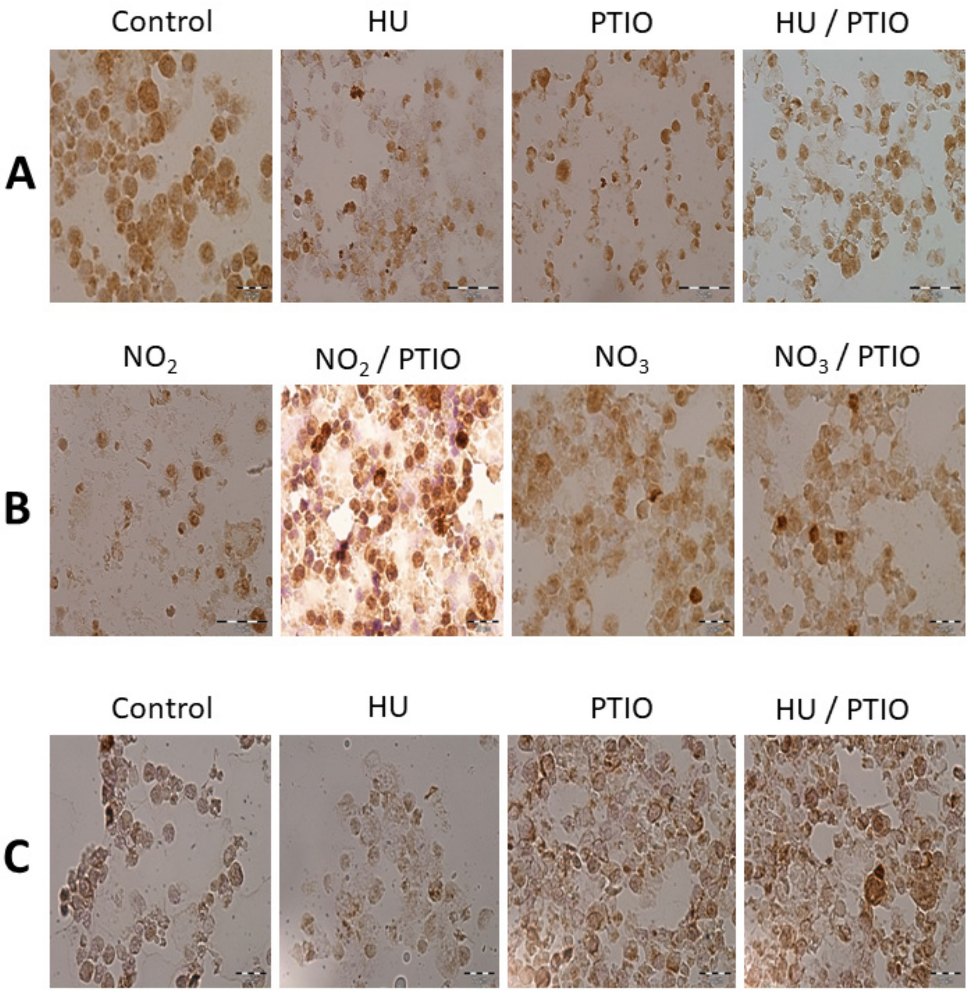

Figure S3. Immunocytochemistry slides correspond to Figure 6. Columns in graphs (**A**,**C**), while in graph (**B**) corresponds to NO_2, NO_2/PTIO, NO_3, NO_3/PTIO labelled columns.
